# Post-radiotherapy osteomyelitis of the cervical spine in head and neck cancer patients

**DOI:** 10.1259/bjro.20230001

**Published:** 2023-09-28

**Authors:** Nir Tsur, Ella Segal, Noga Kurman, Sharon Tzelnick, Ory Wiesel, Lior Wilk, Yaniv Hamzany, Gideon Bachar, Hagit Shoffel-Havakuk

**Affiliations:** 1 Department of Otolaryngology-Head and Neck Surgery, Rabin Medical Center – Beilinson Hospital, Petach Tikva, Israel; 2 Sackler Faculty of Medicine, Tel Aviv University, Tel Aviv, Israel; 3 Department of Radiotherapy, Davidoff Cancer Center, Rabin Medical Center – Beilinson Hospital, Petach Tikva, Israel; 4 Division of Thoracic and Esophageal Surgery, Baruch- Padeh Medical Center, Poriya, Israel; 5 Faculty of Medicine in Galilee, Bar Ilan University, Ramat Gan, Israel; 6 Division of Diagnostic and Interventional Imaging, Soroka, University Medical Center, Beer-Sheva, Israel

## Abstract

**Objective:**

To evaluate patient characteristics, risk factors, disease course, and management of cervical vertebral osteomyelitis in patients who had radiation for head and neck cancers.

**Methods:**

A retrospective cohort study (case series) of patients diagnosed with post-radiation osteomyelitis of the cervical spine between 2012 and 2021. Data were collected from the patient’s medical files.

**Results:**

Seven patients (71% male) with post-radiation cervical osteomyelitis were reviewed. The median patient age was 64 years. The mean interval between diagnosis of osteomyelitis and the first and last radiotherapy course was 8.3 and 4.0 years, respectively. A medical or surgical event preceded the diagnosis in four patients (57%) by a mean of 46.25 days. Common imaging findings were free air within the cervical structures and fluid collection. Four patients recovered from osteomyelitis during the follow-up within an average of 65 days.

**Conclusion:**

Post-radiation osteomyelitis is characterized by a subtle presentation, challenging diagnosis, prolonged treatment, and poor outcome. Clinicians should maintain a high index of suspicion for the long-term after radiotherapy. Multidisciplinary evaluation and management are warranted.

**Advances in knowledge:**

The study describes post-radiotherapy osteomyelitis of the cervical spine, a rare and devastating complication. Literature data regarding this complication are sparse.

## Introduction

Head and neck cancer (HNC) is the seventh most common malignancy worldwide, with approximately 900,000 new cases and half a million deaths annually.^1,2^ Squamous cell carcinoma is the most common histological type of HNC, accounting for about 90% of patients. Radiotherapy is the mainstay of treatment, with or without chemotherapy or surgery.^3,4^ Variations in the combination and order of treatments depend on tumor site, histology, stage, patient’s medical history, and preference. However, despite advances in diagnosis and treatment, locoregional recurrence occurs in 15–50% of patients, and up to 27% of recovered patients are at risk of secondary primary head and neck tumors.^5–7^

Radiotherapy to the head and neck often causes immunological and vascular changes to the upper aerodigestive mucosa. As a result, it may induce mucosal ulceration, tissue breakdown, and the formation of non-healing wounds and fistulas, through which microorganisms colonizing the irradiated mucosa can penetrate and infect the soft tissue and bone.^8^ Furthermore, substantially adverse effects may be on cellular physiology, including inhibition of osteoblast and osteoclast activity, vascular injury, cellular metabolic imbalance leading to osteolysis, increased susceptibility to infection, and tissue necrosis.^9^

Osteoradionecrosis (ORN) is when an irradiated bone becomes exposed through a wound in the overlying skin or mucosa and persists without healing for 3–6 months.^10–12^ ORN affects 2% of irradiated patients with HNC, commonly presenting in the mandible and maxillary bones.^13^ ORN of the mandible was described in up to 5% of patients after head and neck irradiation and was attributed to the bone density that absorbs a more considerable amount of radiation and less vascular supply than other bones.^11^ Conversely, ORN of the cervical spine and skull base is a rare condition.^14,15^ As opposed to the mandible and maxilla, the cervical spine and skull base are deeper beyond anatomical barriers and not in close contact with the contaminated biofilm of the oral cavity mucosa; also, the cervical spine and skull base are rarely positioned within the maximal radiation field, as in the case of oral cavity cancer and the jaws.

Cervical vertebrae osteomyelitis refers to the infection and inflammation of the bone and bone marrow in the cervical spine (neck region). It is typically caused by bacteria, such as *Staphylococcus aureus*, which can enter the vertebrae through various routes, including direct trauma, surgery, or bloodstream infections. Osteomyelitis can affect any bone in the body, including the cervical vertebrae. Compared to osteomyelitis, radionecrosis refers to tissue death or damage due to exposure to radiation therapy. It most commonly affects tissues that have been irradiated as part of cancer treatment. Radionecrosis can occur in various body regions, including the head and neck, where the cervical vertebrae are located.^16,17^ To summarize, cervical vertebrae osteomyelitis is an infection and inflammatory condition of the cervical spine bones, usually caused by bacteria. Radionecrosis, conversely, is tissue death or damage resulting from prior radiation therapy, commonly seen in the cervical vertebrae following treatment for HNC.

The treatment of head and neck osteomyelitis may require specific expertise. Besides the tendency for polymicrobial infections, important anatomic considerations are owing to the challenging drainage approach and the proximity of essential major blood vessels and the skull base. In some cases, a prolonged antibiotic regimen may be sufficient, whereas resistant disease often requires additional surgical debridement.^18–21^ In addition, radiation may damage the cervical vertebrae and the adjacent ligamentous structures, which are close to the radiation field, resulting in cervical spine deformity and instability and, ultimately, spinal cord compression, neurological deficit, and myelopathy.^22,23^

Although ORN and osteomyelitis of the cervical spine and skull base are devastating complications of HNC radiation, they have not been thoroughly described in the medical literature. Therefore, this study aims to present a series of those patients and analyze their characteristics, identify potential risk factors, and describe disease course and management.

## Material and methods

A retrospective case series study was conducted in the tertiary radiation oncology center, Davidoff Cancer Center for the Treatment and Research of Cancer of Rabin Medical Center. The institutional ethics committee approved the study protocol (IRB-XXX 0731–2020). The cohort consisted of patients previously treated with radiation, with or without chemotherapy and surgery, for HNC who presented to the institute’s multidisciplinary head and neck boards (either tumor boards or radiology rounds) between 2012 and 2020 with a diagnosis of osteomyelitis of the cervical spine or skull base region based on a combination of clinical findings, radiological features and a culture positive finding from the infection site without another infectious site. Clinical data for the study were collected from the patient’s medical files: demographics, past medical and surgical history, detailed radiation therapy protocols, clinical presentation, imaging characteristics, clinical course, and management of osteomyelitis. The duration of follow-up was calculated in months from diagnosis to the last follow-up visit. Recovery time was calculated in days from diagnosis to resolution of osteomyelitis, as noted in the discharge form or follow-up report.

Categorical variables are presented as absolute values and percentages; continuous variables are presented as median and range.

## Results

**Figure 1.**
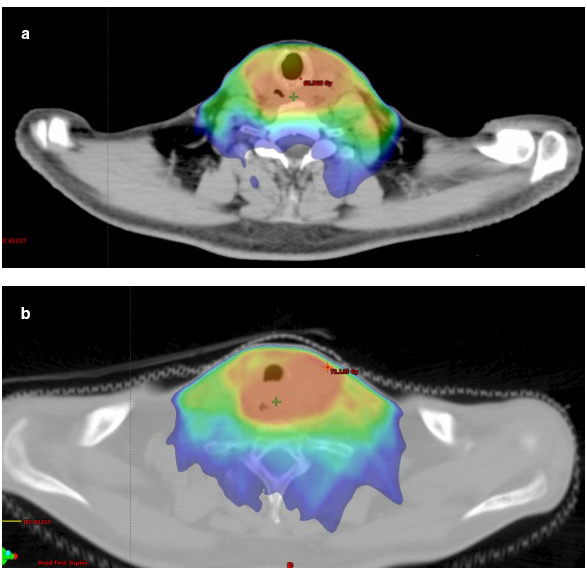
An example of comparative treatment plans using relative dose color wash of axial slices from a 3D conformal portion plan for a patient with nasopharyngeal carcinoma (a) and Glottic SCC (b).

Seven patients were enrolled in the study: five males (71%) and two females of median age of 64 years (range 54–85) at diagnosis. The characteristics and medical history of the patients are detailed in Table 1. The average interval between initiation of steroid treatment and diagnosis of osteomyelitis was 139.5 days (range 28–426 days); Table 2 summarizes the malignant disease and treatment characteristics. Four patients (57%) had squamous cell carcinoma of the head and neck, two had nasopharyngeal carcinoma (29%), and one (14%) had papillary thyroid carcinoma. Most patients were treated with radiochemotherapy (five patients, 71%), and two of them also had surgery. Of the remainder, one patient was treated with radiation alone, and one patient with radiation and surgery. Four patients (57%) were reirradiated for treatment of either recurrence of the primary tumor or second primary HNSCC (head and neck squamous cell carcinoma). The mean interval between completion of the first course of radiotherapy and diagnosis of osteomyelitis was 8.09 years (range 1.69–21.26 years), and between completion of the last period of radiotherapy and diagnosis of osteomyelitis was 4 years (range 0.26–11.34 years). In the subgroup of reirradiated patients, the mean interval between completion of the last course of radiotherapy and diagnosis of osteomyelitis was 1.58 years (range 0.26–4.32 years). Figure 1 shows treatment plans using absolute dose color wash for three patients.

**Table 1.** Patients' characteristics and medical history

Patient number	Gender	Age	Number of comorbidities *	DM	Smoking status	Total pack years	Recent steroid treatment	Days from initiation of steroid treatment to OM	Tracheostomy
1	M	54	8	Yes	AS	40	Yes	426	No
2	M	63	3	No	AS	60	No		Yes
3	F	85	3	No	N	0	Yes, >90, ++	7	No
4	M	64	5	Yes	N	0	No		Yes
5	M	62	2	No	N	0	Yes, >90, ++	28	No
6	F	74	1	No	N	0	Yes, >90	97	Yes
7	M	73	2	No	N	0	No		No

DM, Diabetes Mellitus; OM, Osteomyelitis.

**Table 2.** Malignant disease and treatment characteristics

	Malignancy	TNM-Stage	Treatment modalities	First radiation (Type, dose)	Second radiation (Type, dose)	Third radiation (Type, dose)	Fourth radiation (Type, dose)	Years between the first radiation and OM	Years between the last radiation and OM	Mean Dose at OM site	Max dose at OM site	HBO
1	Glottic SCC	T4aN0M0Stage 4a	CRT	IMRT, 70 Gy	None	None	None	2.78	2.78	48.4	70.1	No
2	Glottic SCC	T1N0M0 Stage 1	RT alone	EBRT	None	None	None	13.18	13.18	14	59.2	No
3	Hypopharynx SCC, PTC	T3N0M0 Stage 3	CRT	EBRT, 70 Gy	None	None	None	5.42	5.42	49.8	64.35	No
4	Glottic SCC, Hypopharynx SCC	T4aN0M0Stage 4a	CRT + Surgery	EBERT	Missing, 70 Gy	None	None	11.90	4.32	47.8	69	No
5	NPC	T2N0M0 Stage 2	CRT	EBRT, 70 Gy	SBRT, 55 Gy	None	None	2.18	0.95	58.1	73	Yes
6	PTC	T4aN0M Stage 3	RT + Surgery	RAI, 150 mci	RAI, 200mci	EBRT, 66 Gy	EBRT, 66 Gy	21.26	0.26	52.1	66.2	No
7	NPC	T4aN1M0Stage 3	CRT + Surgery	SBRT	SBRT	None	None	1.69	0.79	45.1	67.56	No

EBRT, External Beam Radiotherapy.

### Imaging and infectious-related data

**Figure 2.**
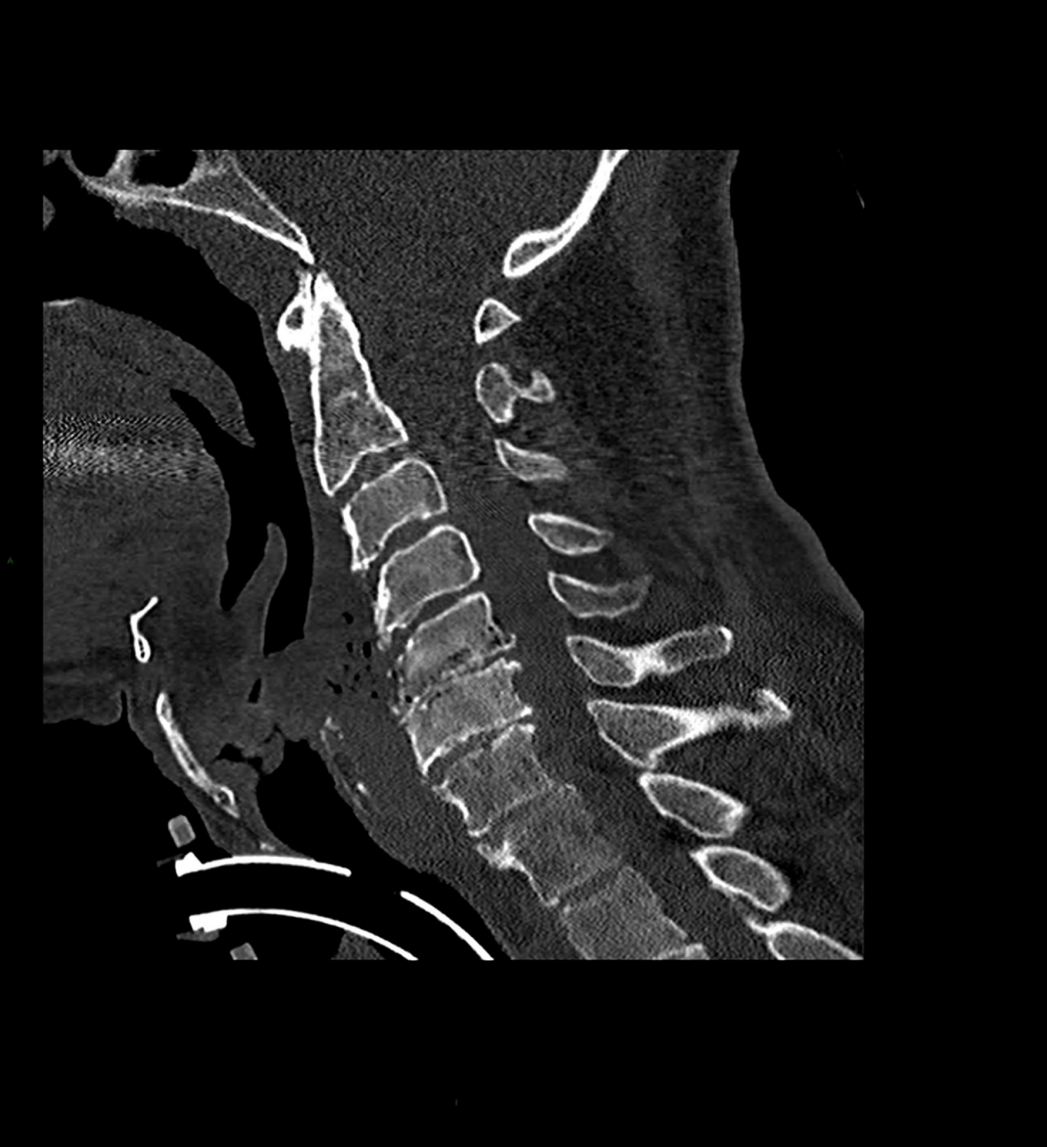
A SAGITAL NCCT of the cervical spine in bone window shows cortical irregularity in the anterior vertebral body of C5 and C6, prevertebral soft tissue swelling with air in the soft tissue and in the spinal canal.

**Figure 3.**
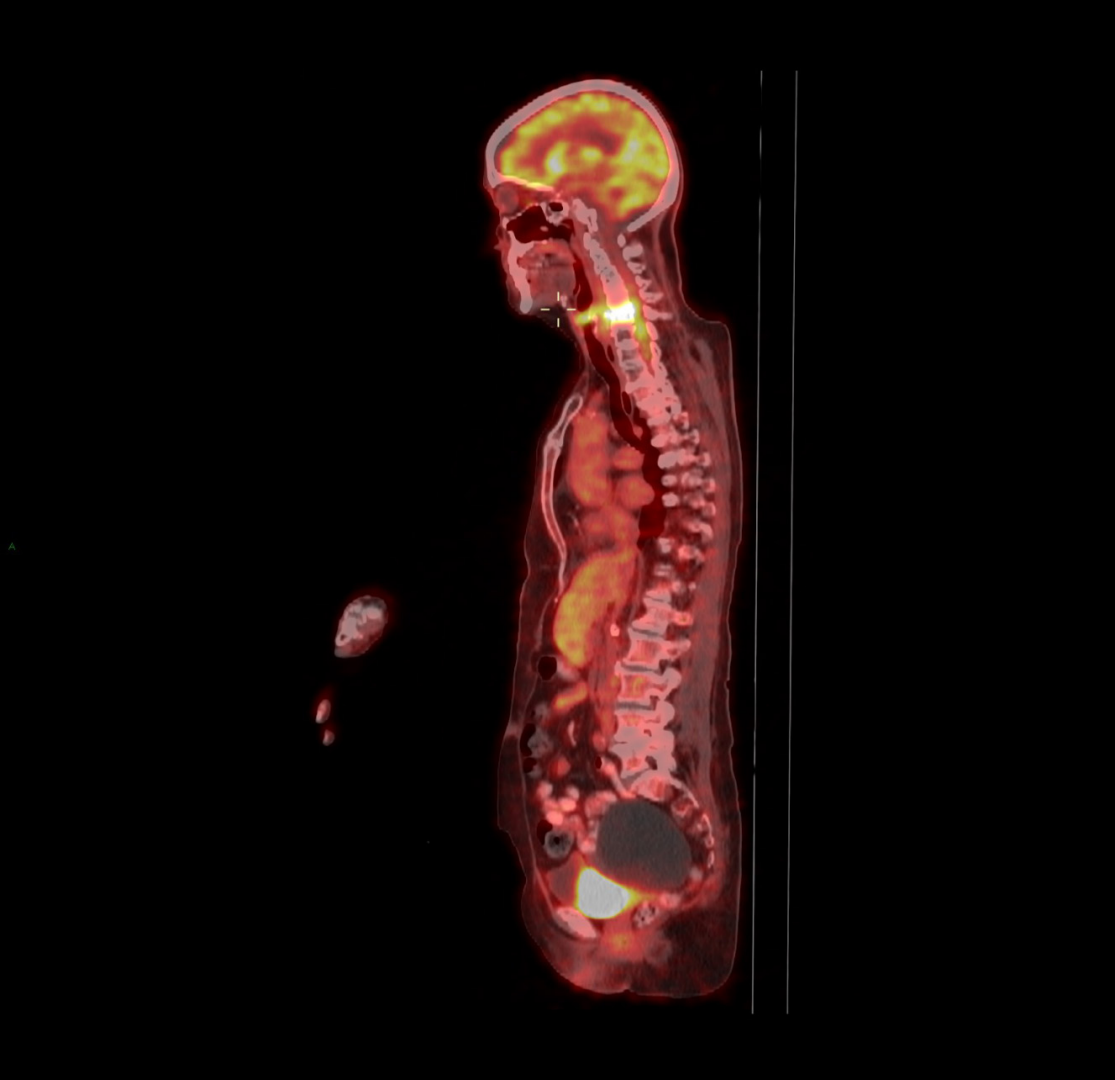
C PET CT show high FDG intake in prevertebral soft tissue and C5-C6 vertebral bodies.

**Figure 4.**
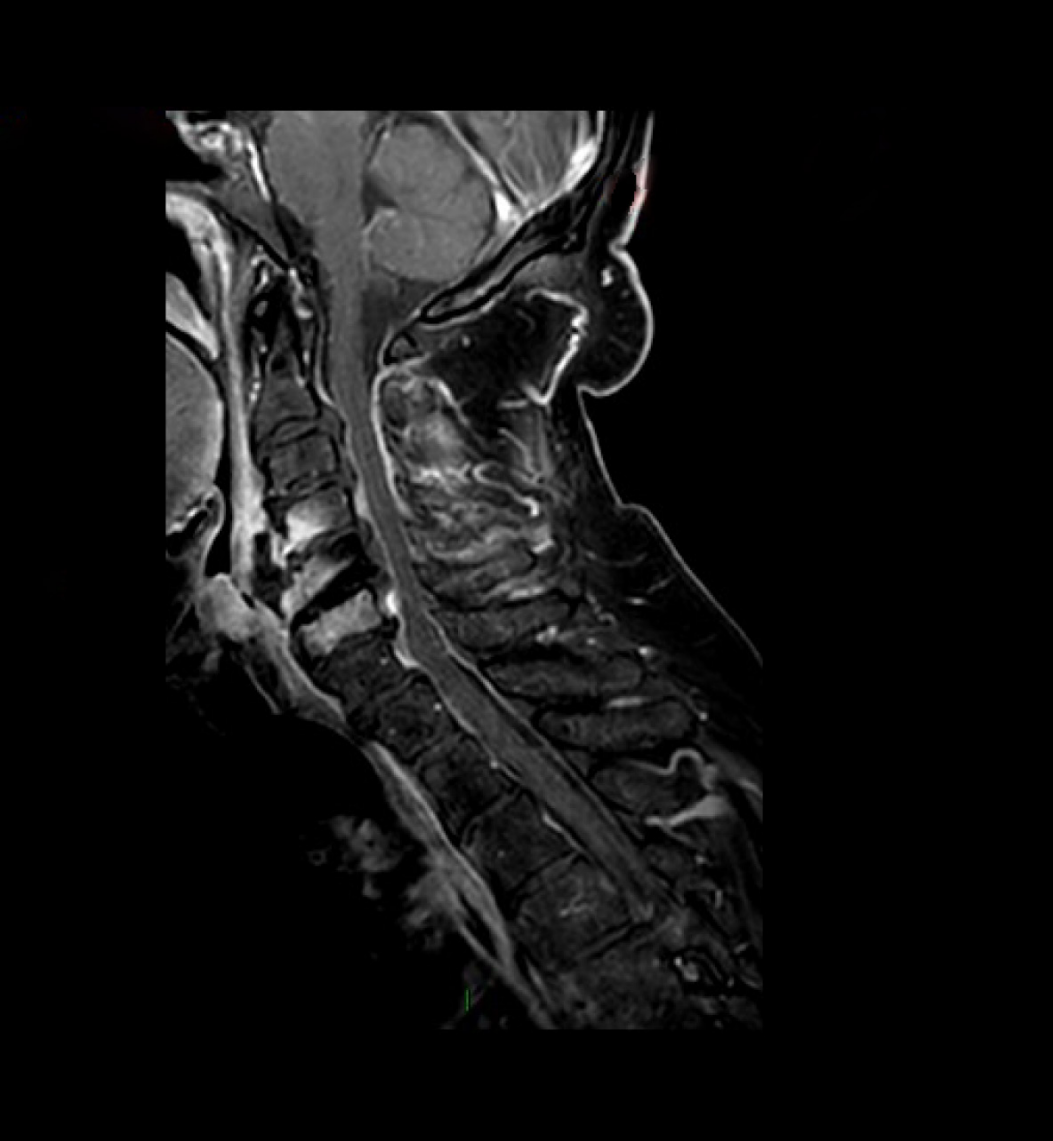
B SAGITAL TIW FS post gadolinium injection of the cervical spine shows enhancement of C4-C6 vertebral bodies, prevertebral and epidural abscess.

**Figure 5.**
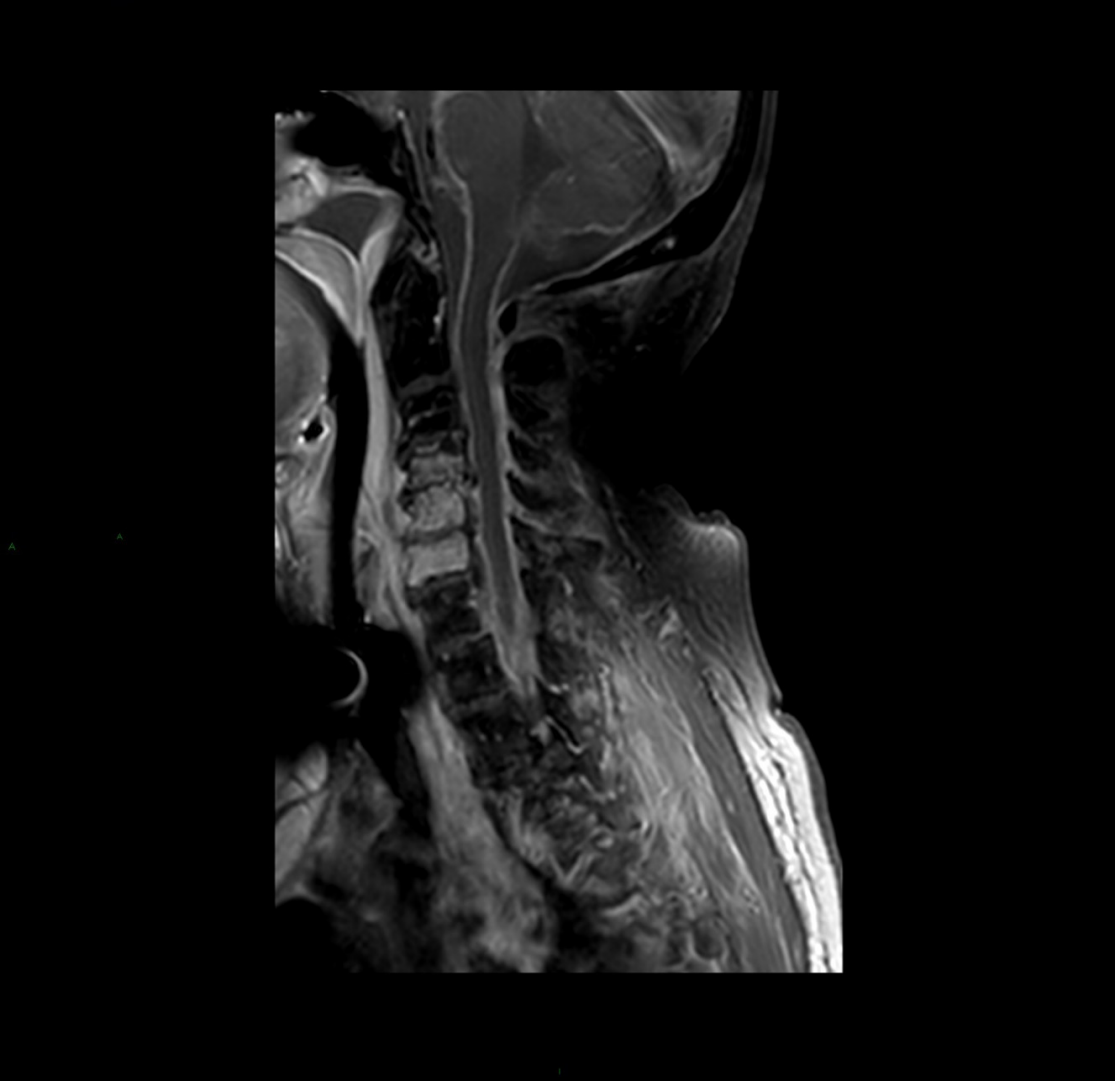
D SAGITAL TIW FS GD+ of cervical spine shows enhancement of C4-C6 vertebral bodies, prevertebral abscess and meningeal enhancement in the posterior fossa and cervical spine.

All patients had imaging studies, as detailed in Table 3. Imaging included CT-non-contrast (NCCT) and contrast enhancement (CECT), MRI, and positron emission tomography-CT (PET-CT). Contrast material enhancement was noted in seven patients (100%): vertebral enhancement in five and meningeal and epidural enhancement in four. Figures 2–5 were carefully collected and described key features of the patients in our series. A microbial culture was held in six patients (85%); results are detailed in Table 4.

**Table 3.** Imaging findings

	Imaging type	Location of OM	Free air	Collection	Enhancement	Spinal pressure	Spinal degenerative disease
1	MRI	C5-C6	Yes	No	Yes	Yes	No
2	MRI	C4-C6	Yes	Yes	Yes	Yes	No
3	PET-CT	C5	Yes	Yes	Yes	No	Yes
4	CT + MRI	C3-6	No	Yes	Yes	No	No
5	PET-CT	Clivus	Yes	No	Yes	Yes	Yes
6	MRI	C7-T1	No	Yes	Yes	No	No
7	PET-CT	Clivus+c1	No	No	Yes	No	Yes

CT, Computed Tomography; MRI, Magnetic Resonance Imaging; OM, Osteomyelitis; PET, positron emission tomography.

**Table 4.** Infectious-related data

	Preceding event	Infecting pathogen by culture	Antibiotic treatment	Surgical debridement or intervention
1	None	1. *Strep. constellatus* 2. *Enterobacter cloacae* 3. *Haem. influenzae* 4. *Strep. anginosus* 5. *Prevotella* spp 6. *Staph. coag*. Neg	Vancomycin + Clindamycin	Debridement
2	None	1. *Staph. capitis* 2. *Strep. mitis* 3. *Neisseria subflava*. 4. *Proteus mirabilis* 5. *Pseud. aeruginosa*, 6. *Strep. viridans*	Missing	Cervical fusion
3	Pneumonia	No	Vancomycin + Rocephin	None
4	Surgical alternative enteral feeding	1. *Coryneb. striatum* 2. *E. coli* 3. *Kleb. pneumoniae*	Meropenem + Vancomycin	Debridement, cervical fusion
5	Surgical alternative enteral feeding	*S. aureus*	Ceftriaxone	None
6	None	*Pseud. aeurginosa, Serratia marcescens, Enterococcus corynebacterium, Neisseria subflava*	Ciprofloxacin + Amocixillin>Tazocin	None
7	Biopsy	*Staph. aureus*	Cefazolin	Cervical fusion

### Presentation, symptoms, findings, and laboratory tests

Symptoms included dysphagia (71%), cervicalgia (71%), weight loss (29%), cough (14%), and hoarseness (14%). Six patients (86%) were presented with multiple symptoms (two or more). On physical examination, fever was present in two patients (29%), posterior pharyngeal mucosal defects was found in three patients (43%), showing an ulcer with exposed bone tissue. Finally, abnormal reflexes in all four limbs were presented in one patient (14%). Initial blood tests revealed leukocytosis in four patients (57%) and an average leukocyte count of 11.14 K ml^−1^ (range 8.06–15.56 K ml^−1^).

### Preceding events and possible triggers

In four patients (57%), a surgical or medical event preceded the diagnosis of osteomyelitis, as detailed in Table 4. These included an invasive intervention in three patients (43%), namely surgical alternative enteral feeding and biopsy, and pneumonia in one patient (14%). The mean interval from the precedent event to the diagnosis of osteomyelitis was 46.25 days (range 6–125 days). For these four patients, the mean interval from completion of the first radiotherapy to the diagnosis of osteomyelitis was 5.30 years (range 1.69–11.9). Three patients had tracheostomies while diagnosed with osteomyelitis. The mean interval between tracheostomy insertion and osteomyelitis diagnosis was 5.30 years (range 0.75–11.18)

### Treatment and outcome

Table 4 shows infectious-related data. Four patients (57%) required surgical intervention and antibiotic treatment for osteomyelitis, and in three (43%), long-term broad-spectrum intravenous antibiotics alone were sufficient. Overall, 86% of patients received broad-spectrum antibiotics. Of the four patients (57%) with cervical spinal instability, three underwent cervical fusion surgery, and one was treated with a cervical collar alone. One patient was treated with hyperbaric oxygen therapy, which comprised 30 decompressions due to anaerobic drug-resistant bacteria. The average duration of follow-up was 1.90 months (range 11 days–3.97 months). Four patients recovered from osteomyelitis; the average time to recovery in the four patients for whom data were available was 65 days (range 18–118 days).

Five patients (71%) died during follow-up; three related to osteomyelitis—two died from septic shock, and one from cervical hemorrhage. Those patients died within 35 days of diagnosis of osteomyelitis (range 11–52 days). The remaining two patients died due to sepsis, unrelated to osteomyelitis: one due to pneumonia and the other urosepsis.

## Discussion

Osteomyelitis of the cervical spine and skull base is an infrequent but dangerous head and neck radiation complication. Sparse data are available in the literature. The present case series describes the characteristics and clinical course of seven affected patients treated in a tertiary medical center over 8 years. Notably, in most patients, a medical or surgical event occurred in the months preceding the diagnosis of osteomyelitis. Multidisciplinary treatment was required, including broad-spectrum antibiotics and surgery. Recovery time was long, and outcomes were poor.

Post-radiation osteomyelitis has a variable presentation.^24^ The mean time in our cohort between the first radiation treatment and diagnosis of osteomyelitis was long and had a wide range, similar to the experience of other centers.^24^ Thus, clinicians should maintain a high index of suspicion even decades after radiotherapy, alongside regular evaluation for recurrent or persistent squamous cell carcinoma.^22^ When post-radiation osteomyelitis is suspected, a thorough evaluation should be conducted, including flexible nasal endoscopy to assess discharge, local swelling, edema, and pharyngeal wall defect, in conjunction with a complete radiological assessment. The radiological assessment may be complex because of significant scar tissue from prior radiotherapy, limiting soft tissue swelling on lateral cervical spine X-rays. MRI is the modality of choice. Hyperintense signals on *T*_2_ weighted images indicate infection and hypointensity on T1 images indicates loss of marrow fat signal.^8,25,26^ Contrast enhancement on T1 may be present with a soft-tissue inflammatory mass or a low-grade infection. However, MRI cannot differentiate infection superimposed on ORN from pure osteomyelitis.

In our study, patients were evaluated using MRI, CT, PET-CT, or a combination of those studies, which yielded findings that might indicate active inflammation, which include contrast material enhancement of the vertebral body epidural and meningeal enhancement, fluid collection, free air caused by gas-forming bacteria, and reactive lymphadenopathy. Thus, several imaging modalities may help diagnose active chronic infection, which would require an aggressive treatment approach. Table 5 addresses the imaging-based differences between osteomyelitis and the most relevant differential diagnosis—osteoradionecrosis based on different modalities of imaging.

**Table 5.** 

Imaging findings	Osteoradionecrosis	Osteomyelitis
**MRI**	1 Decreased T1 signal intensity 2 Increased T2 signal intensity 3 Vertebral collapse or fragmentation 4 Lack of enhancement	5 Bone marrow edema (increased T2 signal intensity) 6 Vertebral destruction 7 Paravertebral soft tissue changes
**CT**	8 Cortical bone thinning or sclerosis 9 Soft tissue changes (edema, fibrosis)	10 Cortical bone erosion or destruction 11 Soft tissue swelling or abscess formation
**Nuclear medicine**	12 Decreased uptake on bone scans 13 Lack of abnormal tracer uptake on PET scans	14 Increased uptake on bone scans (infectious/inflammatory)
**Clinical features**	15 History of prior radiation therapy 16 Delayed onset (months to years after radiation) 17 Progressive symptoms 18 History of malignancy or radiation treatment	19 Infection-related symptoms (fever, chills, elevated WBC) 20 Acute or subacute presentation 21 Pain, tenderness, restricted neck movement

PET, positron emission tomography.

In equivocal cases, bone biopsies can help identify ORN. All patients should undergo biopsies since tumor recurrence is an important differential diagnosis.^22^ Samples should include the suspected bone and surrounding soft tissue. Some authors advocated transoral instead of CT-guided biopsy via an anterior or transverse approach because CT-guided biopsy poses a risk to vital structures like the carotid triangle.^22^ This is especially true in necks that have been irradiated in which the extensive scarring distorts the anatomy.

The treatment of osteomyelitis is complex, let alone osteomyelitis of the head and neck central compartment owing to the complicated anatomy and the proximity to critical vascular, parenchymal structures and skull base. It usually includes surgery in addition to long-term broad-spectrum antibiotics.^27^ In our study, most patients required debridement and cervical fusion. In addition, surgical decompression would likely be necessary in the event of spinal cord compression by an epidural abscess. Furthermore, a few case reports have suggested the possible advantages of hyperbaric oxygen, including abscess reduction and improvement in mucosal defect.^28^ Thus, due to the potential benefits and relatively minor side-effects, hyperbaric oxygen should be considered in those dreadful complications. Further prospective research should better evaluate the advantages of hyperbaric oxygen.

A critical finding in the present study was that a precedent event in most patients may have triggered osteomyelitis. Invasive procedures and localized infections in irradiated patients might cause temporary bacteremia, leading to infection in the damaged tissue. In addition, prior case reports have described anaerobic bacteremia after tracheostomy that might further complicate osteoradionecrotic tissue causing osteomyelitis in the head and neck central tissues.^29^ Nevertheless, we believe routine prophylactic antibiotics are not advised because of the complications of prolonged antibiotic use.

Cervical spine osteomyelitis is a dreadful complication with devastating short- and long-term outcomes causing high degrees of morbidity, debilitation, and mortality.^30^ In our research, the majority of patients who died during follow-up had an osteomyelitis-related death, and others died of severe inflammatory response syndrome due to infection. Cervical spine osteomyelitis is a complication that may cause mortality and indicates a poor patient prognosis since all those patients were malnourished, active, or cured of advanced-stage HNSCC after one or more high-dose radiation treatments with possible concurrent chemotherapy.

Given the subtle presentation, challenging differential diagnosis, long treatment duration, and poor outcomes, we suggest an individually tailored management approach for patients with radiation-induced osteomyelitis, carried out by a multidisciplinary team consisting of a head and neck surgeon, neurosurgeons, orthopedic surgeons, head and neck-oriented radiologist, nuclear medicine expert, infectious disease specialist, and radiological oncologist.

In recent decades, substantial advances have been made in radiotherapy, such as the transition from a two-dimensional to a three-dimensional technique. Additionally, the introduction of stereotactic radiotherapy allows for accurate delivery of high-dose radiation in multiple directions, thereby causing fewer side-effects.^31^ Nevertheless, head and neck radiotherapy poses a high risk of spinal complications requiring careful monitoring of patients.^32–34^

This study was limited, first and foremost, by aa small cohort, single-institution experience, and a comparable control group.In addition, due to the retrospective design, the reported data might be incomplete.

## Conclusion

Midline structure osteomyelitis is a devastating complication of head and neck radiotherapy. Data in the literature remain sparse. Diagnosis may be difficult because symptoms are often delayed and may be subtle. A thorough evaluation, including endoscopic examination, MRI, and possibly biopsy, must be performed in all cases to rule out tumor recurrence or metastasis, and infections should be treated aggressively with antibiotics. Internal stabilization and fusion should be performed in cases of instability and deformity. The present case series highlights the multidisciplinary protocol used in our institute, Rabin Medical Center, to evaluate patients with radiation-induced osteomyelitis to narrow the differential diagnosis, optimize work-up, and initiate prompt appropriate integrative treatment. Its application in various oncology centers worldwide is an attainable goal. We conclude that our initial results merit a continued effort in this direction.
